# Pesticide use and risk of non-Hodgkin lymphoid malignancies in agricultural cohorts from France, Norway and the USA: a pooled analysis from the AGRICOH consortium

**DOI:** 10.1093/ije/dyz017

**Published:** 2019-03-18

**Authors:** Maria E Leon, Leah H Schinasi, Pierre Lebailly, Laura E Beane Freeman, Karl-Christian Nordby, Gilles Ferro, Alain Monnereau, Maartje Brouwer, Séverine Tual, Isabelle Baldi, Kristina Kjaerheim, Jonathan N Hofmann, Petter Kristensen, Stella Koutros, Kurt Straif, Hans Kromhout, Joachim Schüz

**Affiliations:** 1 Section of Environment and Radiation, International Agency for Research on Cancer (IARC), Lyon, France; 2 Department of Environmental and Occupational Health, Drexel University, Philadelphia, PA, USA; 3 ANTICIPE, U1086 INSERM, Université de Caen Normandie, and Centre de Lutte Contre le Cancer François Baclesse, Caen, France; 4 Occupational and Environmental Epidemiology Branch, Division of Cancer Epidemiology and Genetics, National Cancer Institute (NCI), Bethesda, MD, USA; 5 Department of Occupational Medicine and Epidemiology, National Institute of Occupational Health (STAMI), Oslo, Norway; 6 Hematological Malignancies Registry of Gironde, Bergonie Institute, Comprehensive Cancer Centre, Bordeaux, France; 7 University of Bordeaux, INSERM U1219 Center - EPICENE Team, CHU de Bordeaux, Bordeaux, France; 8 Institute for Risk Assessment Sciences (IRAS), Utrecht University, Utrecht, The Netherlands; 9 CHU de Bordeaux, Service de Médecine du Travail et Pathologie Professionnelle, Bordeaux, France; 10 Department of Research, Cancer Registry of Norway, Oslo, Norway; 11 Section of Evidence Synthesis and Classification, International Agency for Research on Cancer (IARC), Lyon, France

**Keywords:** Pesticides, NHL, farmers, cohort, meta-analysis, AGRICOH

## Abstract

**Background:**

Pesticides are commonly used in agriculture, and previous studies endorsed the need to further investigate the possible association between their use and risk of lymphoid malignancies in agricultural workers.

**Methods:**

We investigated the relationship of ever use of 14 selected pesticide chemical groups and 33 individual active chemical ingredients with non-Hodgkin lymphoid malignancies (NHL) overall or major subtypes, in a pooled analysis of three large agricultural worker cohorts. Pesticide use was derived from self-reported history of crops cultivated combined with crop-exposure matrices (France and Norway) or self-reported lifetime use of active ingredients (USA). Cox regression models were used to estimate cohort-specific hazard ratios (HRs) and 95% confidence intervals (CIs), which were combined using random effects meta-analysis to calculate meta-HRs.

**Results:**

During follow-up, 2430 NHL cases were diagnosed in 316 270 farmers accruing 3 574 815 person-years under risk. Most meta-HRs suggested no association. Moderately elevated meta-HRs were seen for: NHL and ever use of terbufos (meta-HR = 1.18, 95% CI: 1.00–1.39); chronic lymphocytic leukaemia/small lymphocytic lymphoma and deltamethrin (1.48, 1.06–2.07); and diffuse large B-cell lymphoma and glyphosate (1.36, 1.00–1.85); as well as inverse associations of NHL with the broader groups of organochlorine insecticides (0.86, 0.74–0.99) and phenoxy herbicides (0.81, 0.67–0.98), but not with active ingredients within these groups, after adjusting for exposure to other pesticides.

**Conclusions:**

Associations of pesticides with NHL appear to be subtype- and chemical-specific. Non-differential exposure misclassification was an important limitation, showing the need for refinement of exposure estimates and exposure–response analyses.

**Key Messages**

- In this analysis combining data from >300 000 farmers or agricultural workers from France, Norway and the USA, accruing more than 3.5 million person-years under risk, the majority of the hazard ratios observed suggested no association of 14 selected pesticide chemical groups and 33 individual active ingredients with the risk of non-Hodgkin lymphoid malignancies (NHL).
- Moderately elevated hazard ratios were seen for NHL overall or certain subtypes with ever use of a few specific pesticides compared with never use of those pesticides: NHL overall and terbufos; chronic lymphocytic leukaemia/small lymphocytic lymphoma (CLL/SLL) and deltamethrin; and diffuse large B-cell lymphoma (DLBCL) and glyphosate; as well as inverse associations of NHL overall with the broader groups of organochlorine insecticides and phenoxy herbicides, after adjusting for exposure to other pesticides. Future work is needed to further investigate these findings.

## Introduction

Pesticide exposure is common in agricultural settings, particularly among farmers and farm workers. Farmers’ activities include multiple tasks that may contribute to exposure, including mixing/loading chemicals, treating seeds, applying in the fields before and during cultivation, re-entry tasks in treated fields, cleaning spraying equipment, protecting crops during storage, controlling ectoparasites in livestock and eradicating insects and parasites in soil, barns and animal compounds.^1–6^ The many pesticide active ingredients used in farming include different compounds that exert their biological effects through diverse mechanisms of action. Therefore, individual active ingredients should also be investigated over broadly defined groups of pesticides. Pesticide exposure can induce genotoxicity, immunosuppression, oxidative stress and/or inflammatory effects, hormone receptor modulation and/or other biological responses that are important characteristics of carcinogens.^7–11^

Chronic exposure to pesticides may partially explain excess incidence and mortality rates of haematological cancer previously reported in farmers.^12^^,^^13^ This group of malignancies encompasses a range of aetiologically distinct subtypes, implying that researching the association of pesticides at the disease subtype level is essential.^14–17^ Yet individual studies may not have enough exposed cases to assess active ingredient–disease subtype associations.^17–19^

AGRICOH is a consortium of agricultural cohort studies [http://agricoh.iarc.fr].^20^ The present study uses data from three large cohorts in the consortium, to explore the relationship between use of selected chemical groups of pesticides and individual active ingredients and risk of non-Hodgkin lymphoid malignancies (NHL) overall and for major subtypes, in 316 270 farmers and farm workers. Chemical groups and active ingredients were selected a priori based on common use in at least two of the three countries, giving preference either to those with some evidence for an association with lympho-haematological malignancies or to agents not previously studied in observational epidemiological studies.

## Methods

### Population

A detailed description of the study population and approaches to harmonizing pesticide exposure data across cohorts is published elsewhere.^21^ Our analysis is based on three cohort studies in the consortium which met the following conditions at the conception of the project in 2012: periodic data linkage to cancer incidence registries, availability of data on pesticides and/or crop cultivation to estimate exposure and sufficiently large sample size to study NHL subtypes.

#### Agriculture and cancer (AGRICAN)

In 2005–07, AGRICAN enrolled 181 747 men and women^22^ who were ≥ 18 years old, affiliated for ≥ 3 years with the national health insurance system of agricultural workers (Mutualité Sociale Agricole) and in 2005 resided in one of 11 departments in France covered by population-based cancer registries. AGRICAN enrolled active and retired farm owners and farm workers as well as some non-farmers, not included in the present analyses. Eligible members were enrolled when returning self-administered questionnaires covering historical information on cultivating 13 crops and raising 5 animal species (including the first and last year of production) and performance of pesticide treatment tasks (the first and last year performed per crop), among other data. Linkage of cohort members with cancer and mortality registries and the National Death Index was done up to 31 December 2009 for this study.

#### Cancer in the Norwegian Agricultural Population (CNAP)

CNAP comprises farm holders (owners and non-owners using a farm) and their families included in at least one of five national agricultural and horticultural censuses conducted in 1969, 1974, 1979, 1985 and 1989 by Statistics Norway.^23^ Only farm holders (male and female) were eligible for inclusion in the present analysis (*n* = 147 134). Census data include type of crops and livestock produced the year before the census, acreage, technology, pesticide expenses and pesticide spraying equipment. Information on crops cultivated, provided in responses to repeated cross-sectional censuses, was used to reconstruct lifelong crop production. Unique personal identification numbers assigned to residents in Norway allowed linkage with the national Cancer Registry of Norway until 31 December 2011, for this study.

#### Agricultural Health Study (AHS)

AHS enrolled 52 394 private pesticide applicators (farmers) and 4916 commercial pesticide applicators registered to apply restricted-use pesticides in Iowa and North Carolina, USA.^24^ Cohort members were enrolled in 1993–97 and completed questionnaires detailing agricultural practices, crops and livestock produced and use of >50 individual pesticide active ingredients. Approximately 5 years later, applicators completed another questionnaire that ascertained pesticide use since enrolment. The present analysis includes only private pesticide applicators (farmers). Since enrolment, cohort members have been regularly linked to the Iowa and North Carolina cancer and mortality registries and the National Death Index. This study includes linkages until 31 December 2011 in Iowa and 31 December 2010 in North Carolina.

Each of the cohort studies contributing to the analysis received institutional review and ethical approval. In addition, the IARC Ethics Committee reviewed and approved the development of the pooling project in October 2012 (Number 12–28).

### Pesticides investigated

At the start of the study, we compiled a list of active ingredients frequently used in at least two of the three countries, giving priority to chemical groups and active ingredients with some evidence for an association with lympho-haematological malignancies, based on the scientific literature,^25^ or to active ingredients not previously investigated in epidemiological studies.

Fourteen chemical groups were included: four groups of insecticides (organophosphates, organochlorines, carbamates and pyrethroids), seven groups of herbicides (phenyl ureas, chloroacetanilides, dinitroanilines, phenoxys, thiocarbamates, triazines,and triazinones), two groups of fungicides (dithiocarbamates and phthalimides),and arsenical compounds, which can have different target pests. Two herbicides that did not belong to any of the chemical groups with other pesticides under investigation (dicamba and glyphosate) were also evaluated. In the three cohorts, a varying number of active ingredients defined exposure at the chemical group level (for a full listing, see Supplementary Table 1, available as Supplementary data at *IJE* online), and only a small number of active ingredients within each chemical group was evaluated individually. Specifically, our evaluation included 33 individual active ingredients, of which two did not belong to the 14 chemical groups examined, listed under Results.

### Exposure assessment

Data from AGRICAN and CNAP were crossed with country-specific crop-exposure matrices (CEMs) to derive estimates of ever exposure to pesticide chemical groups and individual active ingredients.^21^ Among AHS cohort members, self-reported ever application of individual active ingredients was used to assess ever exposure.

#### Crop-exposure matrices

In AGRICAN and CNAP, CEMs were used for a varying number and type of crops: corn, grains, potatoes, vineyards, fruit orchards, tobacco and grassland/meadows in AGRICAN and grains, potatoes, fruit orchards, hay/meadows and greenhouses in CNAP. In AGRICAN, information available in the French PESTIMAT matrix was used, including the first and last year in which each chemical group and active ingredient selected for the study was authorized and recommended for use on a given crop.^26^ For CNAP, matrices were built and provided the first and last year in which each chemical group or active ingredient was sold in the country and authorized for use on each of the selected crops. The CEMs extended from 1950 until the last year of cancer follow-up. In both countries, the CEMs were used to estimate potential exposure to pesticide chemical groups and active ingredients, with cells filled with ever/never (potential) use.

#### Assignment of potential pesticide exposure based on CEM

In AGRICAN, a participant was considered potentially exposed to an active ingredient if: they cultivated a given crop; they marked in the study questionnaire having treated the crop with pesticides; and the active ingredient was authorized and recommended for use on the crop during a given year. In CNAP, a farm holder was considered potentially exposed to an active ingredient if: they reported to have cultivated a given crop; they possessed spraying equipment or spent money on pesticides; and the active ingredient was sold in the country and registered for use on the crop during a given year.

### Follow-up and cancer ascertainment

The endpoint was the first incident NHL during follow-up (i.e. subjects did not have any previous cancer except possibly non-melanoma skin cancer before or during follow-up).In AGRICAN and AHS, the start of follow-up corresponded to the date of enrolment. In CNAP, follow-up started on 1 January 1993, the earliest year of follow-up in one of the other two cohorts (AHS). Follow-up time was calculated as the number of days between the start of follow-up and the first date of the following: (i) first incident cancer (except non-melanoma skin cancer); (ii) loss to follow-up or migration out of the cancer registry area; (iii) death; or (iv) end of follow-up.

Lymphoid malignancies were grouped according to the 2001 WHO classification of haematopoietic tumors and the International Classification of Diseases for Oncology, third edition (ICD-O-3). Each entity was grouped according to the International Lymphoma Epidemiology Consortium.^27^ Here we report findings for non-Hodgkin lymphoid malignancies overall and for the most common subtypes of mature B-cell NHL, including chronic lymphocytic leukaemia/small lymphocytic lymphoma (CLL/SLL), diffuse large B-cell lymphoma (DLBCL), multiple myeloma/plasma-cell leukaemia (MM) and follicular lymphoma (FL).

### Statistical methods

Missing data in AGRICAN (crop, pesticide treatment task, period of production and period of pesticide treatment task) and in AHS (pesticides applied) were imputed five times using the methodology described in White *et al.* (2011)^28^ and Heltshe *et al.* (2012),^29^ respectively. Datasets were subsequently combined using Rubin’s rule (1987).^30^ No imputations were needed for CNAP because exposure data were derived from compulsory agricultural censuses and the information collected on participating farm holders was complete.

Cox proportional hazards models were used to estimate minimally and fully adjusted hazard ratios (HRs) with 95% confidence intervals (CIs) of incident NHL for ever use of the active ingredient or chemical group. Covariates were modelled as time-independent. All values were rounded to the second decimal.

The reference category in all analyses contained participants classified as never exposed to the particular chemical group or specific active ingredient being evaluated. All models used age at the date of censoring as the time scale and were first adjusted for sex and animal production. Additional covariates included retirement status for AGRICAN and state of residence (Iowa or North Carolina) for AHS. Subsequently, fully adjusted models were built separately for each cohort. In AGRICAN, adjustment for the number of crops the farmer/worker reported personally treating with pesticide was added as an ordinal variable indicating increasing opportunity of pesticide exposure. Because similar information was not available in CNAP, adjustment for specific pesticides was used instead. For CNAP and AHS, adjustment for individual pesticides was done using a cohort-specific set of active ingredients (see Tables with HRs). Models were run individually for each cohort, and the resulting estimates were combined using random effects meta-analysis. The I^2^ statistic was used to determine the percentage of the total variance associated with each meta-estimate explained by heterogeneity across cohorts. Acknowledging the large number of comparisons, we report the association between all of the pre-selected 14 chemical groups and 33 active ingredients with NHL overall and the four most frequent subtypes. The statistical software SAS (version 9.4) was used for data management and STATA (version 14) for data analyses.

## Results

### Study population

**Figure 1.**
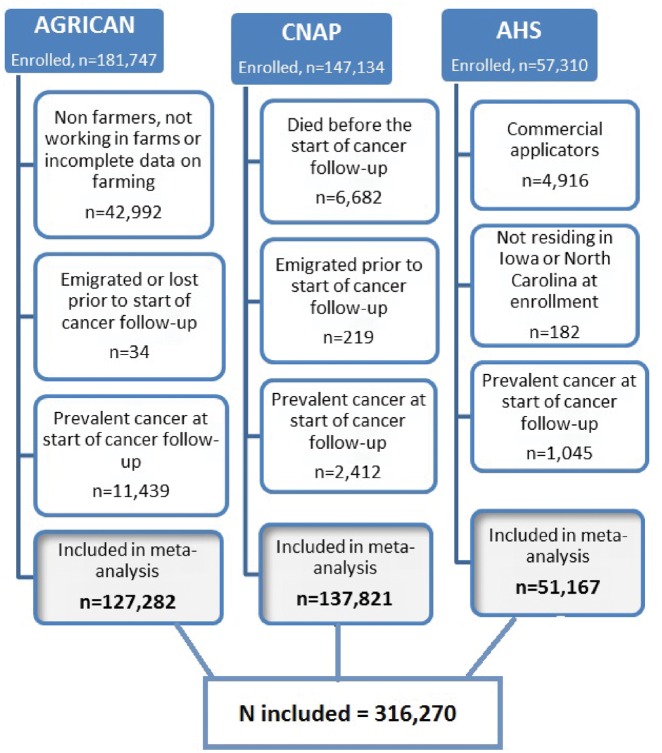
Cohort-specific exclusions and resulting study population included in the meta-analysis.

AGRICAN (127 282), CNAP (137 821) and AHS (51 167) contributed 316 270 participants (Figure 1 ), of whom 237 317 were male (75%). The combined study population accrued 3 574 815 person-years of follow-up from 1 January 1993 until 31 December 2011 (Table 1 ), with a median follow-up of 16 years. During follow-up, 2545 first incident lymphoid cancers were observed (including Hodgkin lymphoma), of which 95.4% (2430) were NHL, with a median age at diagnosis of 69 years (range, 26–98 years). AGRICAN, CNAP and AHS contributed 18.1%, 61.6% and 20.3% of cases of NHL, respectively (details in Supplementary Table 2, available as Supplementary data at *IJE* online).

**Table 1.** Description of study population

Attribute	Combined population		AGRICAN		CNAP		AHS	
	*n*	%	*n*	%	*n*	%	*n*	%
Males	237 317	75%	71 358	56%	116 128	84%	49 831	97%
Females	78 953	25%	55 924	44%	21 693	16%	1336	3%
Both sexes	316 270	100%	127 282	100%	137 821	100%	51 167	100%
Year of birth (range)	1900–1985		1900–1985		1925–1971		1901–1983	
Follow-up period	1993–2011		2005–2009		1993–2011		1993–2011	
Median age at start of cancer follow-up	55		67		51		46	
Median duration of cancer follow-up (years)	16		4		19		16	
Person-years of follow-up	3 574 815		426 340		2 396 595		751 880	
Median age at diagnosis	69		76		68		67	
Participants classified as pesticide applicators or users, based on self-report of pesticide application, performance of pesticide treatment tasks, purchase of pesticides and/or owning spraying equipment	224 060	71%	86 509	68%	87 009	63%	50 542	99%
Prevalence of reported or estimated ever use of at least one of the evaluated chemical groups or active chemical ingredients in the meta-analysis	198 492	63%	80 898	67%	62 047	45%	50 542	99%
Participants who cultivated selected crops^a^								
Hay, meadows, grasslands	142 886	45%	89 168	70%	34 656	25%	19 062	37%
Grains	123 643	39%	73 774	58%	34 838	25%	15 031	29%
Corn	92 861	29%	54 815	43%	n.s.		38 046	74%
Fruit orchards	58 847	19%	49 743	39%	7683	6%	1421	3%
Potatoes	97 705	31%	52 025	41%	43 458	32%	2222	4%
Vineyards	57 815	18%	57 160	45%	n.s.		655	1%
Tobacco	26 203	8%	17 730	14%	n.s.		8473	17%
Greenhouses	23 719	7%	n.s.		23 719	17%		
Soybeans^b^			n.s.		n.s.		36 281	71%
Mean number of selected crops cultivated	2		3.1		1.6		1.1	
Ever producing any animal species	242 695	77%	107 505	84%	102 578	74%	32 612	64%
Lymphoid malignancies diagnosed during follow-up^c^								
Non-Hodgkin lymphoid malignancies (NHL)	2430	100%	439	100%	1498	100%	493	100%
Chronic lymphocytic leukaemia/small lymphocytic lymphoma (CLL/SLL)	497	20.4%	94	21.4%	280	18.7%	123	24.9%
Diffuse large B-cell lymphoma (DLBCL)	434	17.8%	75	17.1%	246	16.4%	113	22.9%
Follicular lymphoma (FL)	214	8.8%	34	7.7%	116	7.7%	64	13.0%
Multiple myeloma/plasma-cell leukaemia (MM)	561	23.5%	103	23.5%	362	24.2%	96	19.5%

n.s., crop not selected for study in the respective country.

^a^In AGRICAN, counts are based on ever cultivating the crop, and these are the average of five imputation datasets, calculated using Rubin’s rule for combining multiple imputed data. Corn includes corn produced for grain or silage, grains include wheat or barley and orchard crops include apples. In CNAP, counts are based on reports of ever producing the crop in any of the agricultural and horticultural censuses (1969–89). In this cohort, no information is available on grasslands, hay or meadows, and the counts reported correspond to silage, used as proxy for grasslands. Grains include wheat, barley, oats, rye and oil seeds, orchard crops include apples, pears and plums, and greenhouses include all crops cultivated in greenhouses. In AHS, counts are based on reports of ever producing the crop at phase I or phase II. Grasslands and meadows include hay and alfalfa, corn includes field and seed corn, and grains include wheat, barley, oats and rye. Vineyards refer to grape cultivation, and orchard crops include apples and peaches. No information on crops cultivated in greenhouses was available.

^b^This crop was not included in the French or Norwegian crop-exposure matrices. Nevertheless, it is included here to give a more complete description of the crops cultivated in the AHS cohort.

^c^Full distribution of lymphoid malignancies is given in Supplementary Table 2, available as Supplementary data at *IJE* online. Percentages of subtypes shown are derived from NHL cases.

Study populations differed by cohort (Table 1). At the start of follow-up, AHS cohort members were younger (median age, 46 years) than those in CNAP (51 years) and AGRICAN (67 years). Approximately half of the AGRICAN cohort (51%) consisted of retired farm owners and farm workers, and 44% of AGRICAN participants were female, compared with only 16% in CNAP and 3% in AHS. In AHS, nearly all cohort members (99%) used pesticides; this percentage was 68% in AGRICAN and 63% in CNAP.

Of the combined population, 63% was classified as ever exposed to at least one of the assessed chemical groups and active ingredients (Table 1; Supplementary Figure 1, available as Supplementary data at *IJE* online shows exposure prevalence by cohort). The list of active ingredients used to determine prevalence of ever exposure to the chemical groups for each cohort is shown in Supplementary Table 1, available as Supplementary data at *IJE* online.

### Risk analyses

Results based on fully adjusted meta-hazard ratios (mHRs) are summarized below for NHL overall and by subtype. Results based on minimally adjusted mHRs are given in Supplementary Table 3, available as Supplementary data at *IJE* online.

### Non-Hodgkin lymphoid malignancies overall

Most of the analyses showed a lack of association with ever use of any chemical group or active ingredient (Table 2 ), with some exceptions. The mHR estimate of association of NHL with ever use of terbufos, based on AGRICAN and AHS (terbufos was not registered for use on the selected crops in Norway), was elevated and with no evidence of heterogeneity of effects between cohorts (mHR = 1.18, 95% CI: 1.00–1.39; I^2 ^= 0%). The cohort-specific HRs for ever use of terbufos and NHL were HR = 1.09 (95% CI: 0.80–1.47) in AGRICAN (96 exposed cases) and HR = 1.22 (95% CI: 1.00–1.49) in AHS (203 exposed cases). Estimated prevalence of ever use was 21% in AGRICAN and 37% in AHS.

**Table 2.** Ever use of 14 pesticide chemical groups and 33 active ingredients and meta-risk estimates, fully adjusted, of non-Hodgkin lymphoid malignancies diagnosed during follow-up in farmers and farm workers in three cohort studies from France, Norway and the USA

	Non-Hodgkin lymphoid malignancies (NHL)					Chronic lymphocytic leukaemia/small lymphocytic lymphoma (CLL/SLL)					Diffuse large B-cell lymphoma (DLBCL)				
	*N* ^a^	HR^b^	95% CI	I^2^	*P* ^e^	*N* ^a^	HR^b^	95% CI	I^2^	*P* ^e^	*N* ^a^	HR^b^	95% CI	I^2^	*P* ^e^
OP Insecticides	1417	0.90	(0.75–1.09)	0%	0.56	318	1.03	(0.64–1.65)	0%	0.65	256	0.90	(0.58–1.39)	0%	0.71
Chlorpyrifos	453	0.99^d^	(0.86–1.15)	0%	0.51	106	1.05^d^	(0.77–1.42)	0%	0.84	86	0.99^d^	(0.72–1.36)	0%	0.76
Dichlorvos	523	1.10	(0.95–1.27)	0%	0.72	116	1.07	(0.78–1.46)	0%	0.39	81	0.94	(0.67–1.32)	0%	0.84
Malathion	1208	0.98	(0.82–1.16)	0%	0.54	274	1.11	(0.76–1.64)	0%	0.89	209	0.84	(0.58–1.21)	0%	0.86
Parathion	995	0.95	(0.81–1.11)	0%	0.58	210	0.99	(0.70–1.40)	0%	0.93	162	0.91	(0.63–1.31)	0%	0.92
Terbufos	300	1.18^d^	(1.00–1.39)	0%	0.53	76	1.28^d^	(0.92–1.79)	0%	1.00	56	1.08^d^	(0.74–1.57)	0%	0.57
Carbamate insecticides	1254	0.94	(0.81–1.08)	0%	0.97	283	1.06	(0.75–1.48)	0%	1.00	219	0.79	(0.56–1.11)	0%	0.77
Aldicarb	526	1.09	(0.95–1.26)	0%	0.67	116	1.16	(0.84–1.60)	0%	0.93	82	0.88	(0.63–1.24)	0%	0.91
Carbaryl	651	0.95	(0.83–1.10)	0%	0.52	148	0.97	(0.71–1.34)	0%	0.67	125	0.87	(0.64–1.19)	0%	0.96
Carbofuran	254	1.00^d^	(0.84–1.20)	0%	0.59	63	1.08^d^	(0.75–1.56)	0%	0.40	44	0.84^d^	(0.55–1.27)	0%	0.92
Pirimicarb	796	0.90^c^	(0.74–1.08)	0%	0.95	176	1.18^c^	(0.77–1.82)	0%	0.51	123	0.71^c^	(0.44–1.14)	0%	0.64
OC insecticides	1258	0.86	(0.74–0.99)	0%	0.77	288	1.08	(0.79–1.48)	0%	0.83	215	0.79	(0.52–1.20)	35%	0.21
DDT	976	0.92	(0.81–1.04)	0%	0.71	220	1.00	(0.77–1.31)	0%	0.75	165	0.85	(0.63–1.14)	0%	0.74
Lindane	953	0.89	(0.78–1.01)	0%	0.87	217	1.06	(0.79–1.41)	0%	0.60	160	0.94	(0.68–1.30)	0%	0.47
Pyrethroid insecticides	934	1.04	(0.90–1.19)	2%	0.36	211	1.29	(0.90–1.84)	15%	0.31	153	0.92	(0.66–1.28)	0%	0.72
Deltamethrin	627	1.04^c^	(0.82–1.33)	59%	0.12	148	1.48^c^	(1.06–2.07)	0%	0.35	99	0.96^c^	(0.68–1.37)	0%	0.64
Esfenvalerate	549	1.15	(0.82–1.63)	69%	0.04	124	1.24^c^	(0.79–1.94)	36%	0.21	89	1.03^c^	(0.71–1.50)	0%	0.93
Permethrin	812	1.01	(0.87–1.19)	0%	0.97	182	1.30	(0.85–2.00)	25%	0.26	131	0.89	(0.61–1.29)	0%	0.96
(Phenyl) urea herbicides	979	1.03	(0.89–1.19)	0%	0.48	209	1.07	(0.75–1.51)	0%	0.97	156	0.86	(0.61–1.21)	0%	0.87
Isoproturon	453	1.03^c^	(0.78–1.35)	55%	0.14	109	1.19^c^	(0.79–1.78)	0%	0.54	73	0.82^c^	(0.49–1.38)	21%	0.26
Linuron	961	1.06	(0.90–1.24)	0%	0.46	204	1.05	(0.73–1.49)	0%	0.92	155	0.94	(0.67–1.33)	0%	0.88
Dicamba	815	1.04	(0.90–1.19)	10%	0.33	180	1.05	(0.80–1.38)	0%	0.83	142	0.95	(0.70–1.29)	0%	0.44
Chloroacetanilides	735	1.00	(0.87–1.14)	0%	0.79	161	0.96	(0.68–1.35)	21%	0.28	145	1.08	(0.79–1.47)	0%	0.68
Alachlor	380	0.97^d^	(0.81–1.15)	0%	0.45	85	0.92^d^	(0.43–1.96)	68%	0.07	74	0.87^d^	(0.60–1.24)	0%	0.87
Metolachlor	358	0.99^d^	(0.84–1.17)	0%	0.57	88	1.02^d^	(0.72–1.43)	0%	0.45	71	0.95^d^	(0.64–1.42)	0%	0.86
Dinitroaniline herbicides	506	0.94^d^	(0.78–1.12)	0%	0.34	126	1.07^d^	(0.74–1.56)	0%	0.42	101	0.91^d^	(0.62–1.31)	0%	0.88
Trifluralin	368	0.98^d^	(0.72–1.33)	64%	0.10	90	0.92^d^	(0.60–1.41)	15%	0.28	72	0.90^d^	(0.62–1.32)	0%	0.66
Glyphosate	1131	0.95	(0.77–1.18)	57%	0.10	252	0.92	(0.69–1.24)	0%	0.38	221	1.36	(1.00–1.85)	0%	0.48
Phenoxy herbicides	1204	0.81	(0.67–0.98)	19%	0.29	272	0.89	(0.61–1.31)	0%	0.79	218	0.70	(0.32–1.52)	59%	0.09
2, 4-D	1167	0.88	(0.75–1.04)	0%	0.87	262	0.92	(0.61–1.39)	19%	0.29	212	0.77	(0.46–1.30)	47%	0.15
MCPA	775	0.81^c^	(0.53–1.23)	65%	0.09	163	0.98^c^	(0.54–1.77)	0%	0.83	125				
MCPP	776	0.94	(0.61–1.46)	65%	0.06	163	1.06^c^	(0.57–1.98)	0%	0.63	124				
Thiocarbamate herbicides	1015	0.93	(0.80–1.08)	0%	0.69	225	0.95	(0.63–1.45)	34%	0.22	171	0.88	(0.61–1.25)	0%	0.88
Butylate	265	1.01^d^	(0.85–1.19)	0%	0.52	60	0.96^d^	(0.50–1.85)	56%	0.13	48	0.89^d^	(0.61–1.32)	0%	0.92
EPTC	606	1.04	(0.91–1.19)	0%	0.74	133	1.10	(0.76–1.60)	35%	0.21	101	1.12	(0.81–1.56)	0%	0.69
Triazine herbicides	1165	0.87	(0.74–1.02)	8%	0.34	269	0.85	(0.60–1.20)	0%	0.72	212	0.84	(0.60–1.18)	0%	0.40
Atrazine	546	0.97^d^	(0.79–1.18)	0%	0.99	131	0.96^d^	(0.62–1.49)	0%	0.35	109	0.88^d^	(0.59–1.31)	0%	0.54
Simazine	303	0.92	(0.77–1.09)	0%	0.43	63	0.83	(0.53–1.31)	6%	0.35	54	0.91	(0.61–1.36)	0%	0.65
Triazinone herbicides	971	1.09	(0.85–1.40)	60%	0.08	215	1.22	(0.84–1.77)	21%	0.28	157	1.00	(0.73–1.36)	0%	0.67
Metribuzin	971	1.09	(0.85–1.40)	60%	0.08	215	1.22	(0.84–1.77)	21%	0.28	157	1.00	(0.73–1.36)	0%	0.67
Dithiocarbamate fungicides	961	0.96	(0.83–1.11)	0%	0.96	196	0.86	(0.62–1.19)	0%	0.72	157	0.93	(0.66–1.31)	0%	0.60
Mancozeb	932	0.94	(0.81–1.09)	0%	0.85	193	0.90	(0.64–1.26)	0%	0.66	149	0.87	(0.62–1.21)	0%	0.87
Thiram	264	0.89^c^	(0.73–1.07)	0%	0.35	54	0.82^c^	(0.40–1.66)	50%	0.16	42	0.79^c^	(0.50–1.25)	0%	0.54
Phthalimide fungicides	921	1.10	(0.80–1.52)	57%	0.10	193	1.13	(0.74–1.72)	0%	0.47	144	0.98	(0.65–1.47)	0%	0.94
Captafol	800	1.12^c^	(0.79–1.59)	69%	0.07	162	1.20^c^	(0.71–2.03)	23%	0.25	121	0.93^c^	(0.62–1.41)	0%	0.44
Captan	481	0.90	(0.74–1.10)	0%	0.45	107	0.92	(0.59–1.44)	13%	0.32	74	0.83	(0.52–1.33)	0%	0.68
Arsenicals	272	0.97^d^	(0.79–1.18)	0%	0.71	60	1.04^d^	(0.66–1.63)	0%	0.49	52	1.19^d^	(0.73–1.95)	0%	0.64


	Follicular lymphoma (FL)					Multiple myeloma/plasma-cell leukaemia (MM)				
	*N* ^a^	HR^b^	95% CI	I^2^	*P* ^e^	*N* ^a^	HR^b^	95% CI	I^2^	*P* ^e^
OP insecticides	131	0.78	(0.34–1.76)	2%	0.36	313	0.85	(0.56–1.29)	0%	0.83
Chlorpyrifos	46	1.08^d^	(0.68–1.70)	0%	0.58	97	0.93^d^	(0.66–1.32)	0%	0.73
Dichlorvos	40	1.10	(0.69–1.76)	0%	0.95	111	0.98	(0.70–1.37)	22%	0.28
Malathion	114	1.18	(0.66–2.10)	0%	0.64	269	0.95	(0.67–1.36)	0%	0.69
Parathion	83	1.24	(0.72–2.15)	0%	0.44	230	0.84	(0.57–1.24)	18%	0.30
Terbufos	35	1.33^d^	(0.80–2.20)	0%	0.70	55	1.01^d^	(0.69–1.49)	0%	0.64
Carbamate insecticides	115	1.03	(0.63–1.68)	0%	0.47	290	1.25	(0.89–1.75)	9%	0.33
Aldicarb	38	0.94	(0.56–1.56)	0%	0.79	113	0.91	(0.68–1.22)	0%	0.58
Carbaryl	67	1.27^d^	(0.66–2.43)	27%	0.24	152	1.11	(0.71–1.74)	47%	0.15
Carbofuran	26	0.91^d^	(0.52–1.59)	0%	0.94	57	1.21^d^	(0.83–1.78)	0%	0.70
Pirimicarb	58	0.84^c^	(0.41–1.71)	0%	0.96	185	1.01^c^	(0.70–1.46)	0%	0.68
OC insecticides	114	0.87	(0.54–1.40)	0%	0.82	290	1.03	(0.76–1.40)	0%	0.97
DDT	79	0.78	(0.45–1.37)	38%	0.20	230	0.96	(0.74–1.25)	0%	0.86
Lindane	78	0.92	(0.61–1.38)	0%	0.57	216	0.97	(0.74–1.28)	0%	0.89
Pyrethroid insecticides	81	1.16	(0.75–1.79)	0%	0.87	210	1.07	(0.79–1.46)	0%	0.72
Deltamethrin	46	1.09^c^	(0.64–1.87)	0%	0.77	135	0.93^c^	(0.69–1.24)	0%	0.97
Esfenvalerate	41	1.04^c^	(0.61–1.75)	0%	0.44	120	1.65	(0.61–4.41)	89%	0.00
Permethrin	60	0.79	(0.42–1.50)	26%	0.26	187	1.18	(0.86–1.63)	0%	0.61
(Phenyl) urea herbicides	81	1.09	(0.65–1.82)	0%	0.84	227	1.08	(0.79–1.47)	0%	0.65
Isoproturon	28	0.70^c^	(0.36–1.36)	0%	0.90	100	1.04^c^	(0.72–1.52)	0%	0.78
Linuron	79	1.11	(0.66–1.87)	0%	0.76	224	1.13	(0.81–1.57)	0%	0.78
Dicamba	73	0.81	(0.53–1.23)	0%	0.63	179	1.21	(0.93–1.59)	0%	0.92
Chloroacetanilides	78	1.00	(0.63–1.58)	0%	0.92	147	0.94	(0.71–1.25)	0%	0.88
Alachlor	43	0.86^d^	(0.47–1.56)	0%	0.96	78	1.06^d^	(0.73–1.54)	0%	0.94
Metolachlor	43	1.05^d^	(0.59–1.86)	0%	0.90	71	1.05^d^	(0.71–1.53)	0%	0.71
Dinitroaniline herbicides	57	0.99^d^	(0.56–1.74)	0%	0.71	95	0.82^d^	(0.42–1.61)	69%	0.07
Trifluralin	39	0.80^d^	(0.47–1.35)	0%	0.66	70	0.89^d^	(0.60–1.32)	0%	0.44
Glyphosate	105	0.79	(0.52–1.21)	0%	0.56	240	0.87	(0.66–1.15)	0%	0.95
Phenoxy herbicides	115	1.02	(0.60–1.75)	0%	0.95	257	0.77	(0.49–1.22)	43%	0.18
2, 4-D	113	1.14	(0.67–1.96)	0%	0.91	250	0.90	(0.65–1.26)	0%	0.53
MCPA	60	1.21^c^	(0.53–2.80)	6%	0.30	174	0.69^c^	(0.29–1.64)	64%	0.10
MCPP	59	0.96^c^	(0.25–3.61)	58%	0.13	175	0.82^c^	(0.25–2.67)	82%	0.02
Thiocarbamate herbicides	88	0.81	(0.52–1.26)	0%	0.68	225	0.99	(0.73–1.34)	0%	0.80
Butylate	31	1.06^d^	(0.63–1.77)	0%	0.94	54	1.04^d^	(0.70–1.53)	0%	0.69
EPTC	49	0.86	(0.42–1.73)	51%	0.13	138	1.19	(0.90–1.59)	0%	0.87
Triazine herbicides	115	1.45	(0.62–3.39)	56%	0.10	246	0.82	(0.50–1.35)	56%	0.10
Atrazine	65	1.28^d^	(0.69–2.38)	0%	0.79	108	0.98^d^	(0.62–1.55)	0%	0.54
Simazine	23	0.91	(0.48–1.70)	0%	0.88	66	0.83	(0.52–1.32)	35%	0.22
Triazinone herbicides	86	0.85	(0.54–1.36)	0%	0.41	221	1.20	(0.73–1.97)	51%	0.13
Metribuzin	86	0.86	(0.54–1.36)	0%	0.41	221	1.20	(0.74–1.97)	51%	0.13
Dithiocarbamate fungicides	75	0.88	(0.52–1.49)	0%	0.98	229	1.11	(0.80–1.52)	0%	0.74
Mancozeb	71	0.80	(0.46–1.39)	0%	0.90	222	1.07	(0.78–1.48)	0%	0.64
Thiram	17	0.84^c^	(0.40–1.79)	0%	0.72	58	0.80^c^	(0.40–1.61)	60%	0.12
Phthalimide fungicides	71	0.73^d^	(0.37–1.44)	0%	0.84	216	1.05	(0.71–1.54)	0%	0.81
Captafol	62	1.11^c^	(0.51–2.42)	10%	0.29	192	1.13^c^	(0.76–1.68)	0%	0.45
Captan	36	0.90	(0.47–1.72)	0%	0.59	97	0.73	(0.39–1.37)	48%	0.15
Arsenicals	17					65	0.91^d^	(0.59–1.40)	0%	0.84


AGRICAN: Cox regression adjusted for: sex, livestock, retirement status, number of selected types of crops for which pesticide treatment personally applied.

CNAP: Cox regression adjusted for: sex, livestock, dichlorvos, aldicarb, lindane, DDT, deltametrin, mancozeb, linuron, glyphosate.

AHS: Cox regression adjusted for: sex, state, livestock, terbufos, lindane, DDT, permethrin, dicamba, parathion, carbaryl.

mHR estimates for DLBCL in association with MCPA and MCPP not available because statistical models did not converge.

mHR estimate for FL in association with arsenicals not available because an estimate could be computed in only one (AGRICAN) of three cohorts. OC, organochlorine; OP, organophosphate; I^2^ (I-squared, variation in meta-estimate attributable to heterogeneity).

^a^Number of cancer cases.

^b^Hazard ratio: random effects meta-analysis (fully adjusted models).

^c^Meta-analysis based on AGRICAN and CNAP only. For esfenvalerate, AHS contributed to mHR of NHL and multiple myeloma.

^d^Meta-analysis based on AGRICAN and AHS only.

^e^ *P*-value for heterogeneity.

In contrast, inverse associations were observed for ever use of organochlorine (OC) insecticides (mHR = 0.86, 95% CI: 0.74–0.99; I^2 ^= 0%) and phenoxy herbicides (mHR = 0.81, 95% CI: 0.67–0.98; I^2 ^= 19%), with no or little heterogeneity of effects between cohorts (Table 2). The cohort-specific HRs for ever use of OC insecticides and NHL were HR = 0.92 (95% CI: 0.72–1.18) in AGRICAN, HR = 0.82 (95% CI: 0.63–1.06) in CNAP and HR = 0.83 (95% CI: 0.65–1.07) in AHS, with 306, 624 and 328 exposed cases, respectively. Estimated prevalence of ever use was 65% 39%, and 54%, respectively. Between nine and 13 individual active ingredients were included per cohort in the OC chemical group. The cohort-specific HRs for ever use of phenoxy herbicides and NHL were HR = 0.94 (95% CI: 0.69–1.28) in AGRICAN, HR = 0.65 (95% CI: 0.46–0.92) in CNAP and HR = 0.83 (95% CI: 0.65–1.06) in AHS, with 178, 637 and 389 exposed cases, respectively. Estimated prevalence of ever use was 38%, 41% and 78%, respectively. Between seven and 10 individual active ingredients were considered per cohort in this chemical group.

The associations between exposure to individual active ingredients within these two chemical groups and NHL were of similar magnitude to those seen at the chemical group exposure level, but for the individual active ingredients the CIs included the null value (Table 2).

### Chronic lymphocytic leukaemia/small lymphocytic lymphoma

During follow-up, 497 incident CLL/SLL cases were diagnosed. A moderately elevated mHR estimate of association of CLL/SLL with ever use of deltamethrin was observed (mHR = 1.48, 95% CI: 1.06–2.07; I^2 ^= 0%; Table 2) based on AGRICAN and CNAP combined data (only 16 AHS farmers, none of them cases, applied the insecticide), with no evidence of heterogeneity of effects between cohorts. The cohort-specific HRs for ever use of deltamethrin and CLL/SLL were HR = 1.17 (95% CI: 0.64–2.13) in AGRICAN (51 exposed cases) and HR = 1.65 (95% CI: 1.10–2.46) in CNAP (97 exposed cases). Estimated prevalence of ever use was 51% in AGRICAN and 25% in CNAP.

### Diffuse large B-cell lymphoma

During follow-up, 434 DLBCL cases were diagnosed. Most analyses did not show any associations (Table 2). There was an elevated mHR of DLBCL with ever use of glyphosate (mHR = 1.36, 95% CI: 1.00–1.85; I^2 ^= 0%), with no evidence of heterogeneity of effects among cohorts. The cohort-specific HRs for ever use of glyphosate and DLBCL were HR = 1.06 (95% CI: 0.51–2.19) in AGRICAN, HR = 1.67 (95% CI: 1.05–2.65) in CNAP and HR = 1.20 (95% CI: 0.72–1.98) in AHS, based on 28, 100 and 93 exposed cases, respectively. Glyphosate was used by 36%, 38% and 83% of the farmers and agricultural workers in AGRICAN, CNAP and AHS, respectively.

### Follicular lymphoma

During follow-up, 214 FL cases were diagnosed. None of the chemical groups or active ingredients was associated with FL (Table 2). Moderately elevated mHRs but with wide CIs were observed in association with ever use of a few active ingredients, of which the least imprecise estimate corresponded to terbufos (mHR = 1.33, 95% CI: 0.80–2.20; I^2 ^= 0%), based on AGRICAN and AHS, with no evidence of heterogeneity of effects between cohorts. The cohort-specific HRs for ever use of terbufos and FL were HR = 1.04 (95% CI: 0.27–3.98) in AGRICAN and HR = 1.38 (95% CI: 0.80–2.30) in AHS, with six and 29 exposed cases, respectively.

### Multiple myeloma/plasma-cell leukaemia

During follow-up, 561 MM cases were diagnosed. None of the chemical groups or active ingredients was associated with MM (Table 2). Moderate elevations in the magnitude of mHRs were observed with ever use of a few active ingredients, of which the least imprecise estimate corresponded to dicamba (mHR = 1.21, 95% CI: 0.93–1.59, I^2 ^= 0%), with no evidence of heterogeneity of effects among cohorts. The cohort-specific HRs for ever use of dicamba and MM were HR = 1.30 (95% CI: 0.70–2.43) in AGRICAN, HR = 1.15 (95% CI: 0.79–1.67) in CNAP and HR = 1.28 (95% CI: 0.77–2.13) in AHS, with 40, 92, and 47 exposed cases, respectively.

## Discussion

In this large international study of exposure to 14 pesticide chemical groups and 33 pesticide active ingredients among participants in three agricultural cohorts with >2400 NHL cases occurring in >3.5 million person-years of follow-up, the vast majority of analyses did not suggest an association between ever use of pesticides and NHL overall or with any NHL subtype. However, we observed some weak to moderate associations with NHL overall and with specific subtypes, suggesting that associations vary by disease subtype and by pesticide.

### Terbufos

We observed a positive association between risk of NHL overall and ever use of terbufos, a soil insecticide with nematicide action that can persist from days to months after application, depending on soil conditions and season.^31^ In France, among the crops included in our analysis, terbufos was first registered for use on corn in 1980 and was used until 2006, when it was no longer registered for use. During this period, it was also registered for use on sunflower and rapeseed cultivation. Currently, terbufos is not approved for use in the EU or in Norway [http://ec.europa.eu/food/plant/pesticides/eu-pesticides-database/public/?event=activesubstance.detail&language=EN&selectedID=1923]. In the USA, it was first registered for use on corn in 1974 and subsequently for use on sugar beets (1976) and sorghum (1982), and it is still registered for use on these three crops [https://www3.epa.gov/pesticides/chem_search/reg_actions/reregistration/red_PC-105001_1-Jul-06.pdf; http://www.kellysolutions.com/ia/showproductsbychem.asp?PC_Code=105001&PctStart=0&PctEnd=100&Chemical_Name=Terbufos]. In 2012, terbufos ranked eighth among the most frequently used organophosphate insecticides in the USA, based on pounds weight of active ingredient sold (<1 million) [https://www.epa.gov/sites/production/files/2017-01/documents/pesticides-industry-sales-usage-2016_0.pdf]. The carcinogenicity of terbufos has not been evaluated by the IARC Monographs (programme on the identification of carcinogenic hazards to humans). In 1994, EPA’s Pesticides Program evaluated the carcinogenic potential of terbufos as Group E (evidence of non-carcinogenicity for humans) [http://npic.orst.edu/chemicals_evaluated.pdf].

Our combined estimate confirms the previously observed association between ever use of terbufos and risk of NHL, CLL/SLL and FL reported in AHS,^32^ with estimates indicating increased risk in the ever exposed after taking into account exposure to other pesticides (same length of cancer follow-up as in the present analysis). In that analysis, an exposure–response trend (*P* = 0.05) was observed with increasing lifetime days of exposure in association with SLL/CLL/mantle-cell lymphoma (MCL) (RR_low_ = 1.3, 95% CI: 0.8–2.0, 32 exposed cases; RR_high_ = 1.6, 95% CI: 1.0–2.5, 31 exposed cases).^32^ A recent meta-analysis of five studies in the published literature reported a positive meta-estimate for ever use, but with the 95% CI including the null value [meta-odds ratio (mOR) = 1.07, 95% CI: 0.85–1.36; I^2 ^= 0%].^33^ Terbufos exposure has also been associated with elevated CLL/SLL risk in a case–control study in the USA, which reported an odds ratio of 2.2 (95% CI: 0.7–7.4) but with wide CIs, based on five exposed cases.^18^

### Deltamethrin

Ever application of deltamethrin was positively associated with an elevated mHR for CLL/SLL based on AGRICAN and CNAP. The mHRs for the other subtypes of NHL in association with deltamethrin were very close to 1 and with CIs including the null value. This insecticide is currently registered for use in the EU, Norway and the USA. At its last evaluation by IARC in 1990 (Volume 53), deltamethrin was classified as Group 3 (not classifiable as to its carcinogenicity to humans), with no data from studies of cancer in humans and only a limited number of assays of cancer in rodents available at the time [http://monographs.iarc.fr/ENG/Classification/latest_classif.php]. The 2002 EC review of deltamethrin specified no genotoxic or carcinogenic potential [http://ec.europa.eu/food/plant/pesticides/eu-pesticides-database/public/?event=activesubstance.detail&language=EN&selectedID=1197]. EPA evaluated the carcinogenic potential of deltamethrin in 2003, classifying it as ‘not likely to be carcinogenic to humans’ [http://npic.orst.edu/chemicals_evaluated.pdf]. No studies of cancer in humans in association with this insecticide were identified in the scientific literature.

### Glyphosate

We did not observe an association between risk of NHL overall and ever use of glyphosate, a broad-spectrum herbicide used in agriculture and other settings. There was, however, evidence of heterogeneity of effects among the cohorts (I^2 ^= 57%). Glyphosate was associated with an elevated mHR for DLBCL, and for DLBCL there was no evidence of heterogeneity of effects among the three cohorts. Cohort-specific associations had wide CIs, with only CNAP, which accounted for 45% of the exposed cases, excluding the null value. In CNAP, adjustment for ever use of other pesticides (linuron, aldicarb, mancozeb, DDT, lindane and deltamethrin) generated a fully adjusted HR for ever use of glyphosate of larger magnitude (1.67, 1.05–2.65) than the minimally adjusted estimate (1.26, 0.97–1.65), driven mainly by adjustment for animal production and ever use of DDT (data not shown). Stratification by DDT use, however, produced elevated DLBCL HRs for ever use of glyphosate in both never or ever use of DDT strata [HR = 1.54 (1.04–2.30), never DDT; HR = 1.95 (0.84–4.52), ever DDT], suggesting that the association with glyphosate was not due to concurrent exposure to DDT. Notably, in the meta-analysis, the mHRs for other subtypes of NHL in association with glyphosate were below 1, with CIs including the null value.

Currently, glyphosate is registered for use in the EU, Norway and the USA. The IARC Monographs evaluated the carcinogenicity of glyphosate in 2015 (Volume 112), classifying it as Group 2 A (probably carcinogenic to humans), based on sufficient evidence of carcinogenicity in animals and limited evidence in humans for NHL.^34^ The European Food Safety Authority (EFSA) concluded that glyphosate is unlikely to represent a carcinogenic hazard to humans from dietary exposure to residual pesticide content [https://www.efsa.europa.eu/en/efsajournal/pub/4302]. In 2016 the Joint FAO/WHO Meeting on Pesticide Residues in food concluded that ‘glyphosate is unlikely to pose a carcinogenic risk to humans from exposure through the diet’ [http://www.who.int/foodsafety/jmprsummary2016.pdf]. In 2015, EPA concluded that glyphosate is ‘not likely to be carcinogenic to humans’, with the draft risk assessment released on 18 December 2017 [https://www.epa.gov/pesticides/epa-releases-draft-risk-assessments-glyphosate].

Whereas the lack of association of ever/never use of glyphosate with NHL overall in our analysis is consistent with a recently published analysis from AHS^35^ reporting no association between lifetime days or intensity-weighted lifetime days of glyphosate use and NHL (440 exposed cases), our mHR observed for DLBCL with ever/never use of glyphosate for the three cohorts combined is higher than that for AHS alone. In addition to evaluating different exposure metrics, our analyses using the AHS data differed in several aspects from the recent AHS publication (2017).^35^ First, the AHS publication included 4619 commercial applicators (non-farmers, and thus excluded from our analysis), but excluded 1620 farmers with information on ever use of glyphosate but who did not report frequency of use (eligible for inclusion in our analysis). The follow-up time was longer in the AHS publication (up to 2012 and 2013), and thus more cases were included than in our analysis (130 vs 113 DLBCL cases). Finally, different variables were used to adjust the risk estimates (in our present analysis, we did not adjust for cigarette smoking, alcohol intake, or family history of any cancer but did adjust for animal production and for different pesticide active ingredients from those included in the AHS publication).

In a previously published meta-analysis of six studies with case–control or cohort design reporting on ever use of glyphosate and overall NHL risk, including a previous publication from AHS that also showed no association with NHL,^36^ a meta-relative risk (mRR) of 1.5 (95% CI: 1.1–2.0) was seen.^25^ Another meta-analysis on glyphosate also reported a positive meta-RR for NHL (1.30, 95% CI: 1.0–1.6; I^2 ^= 13%).^37^

### Organochlorine (OC) insecticides

In contrast to previously published reports suggesting elevation in risk, our analysis found inverse associations between exposure to the broad grouping of OC insecticides and NHL.^16^^,^^38^^,^^39^ There was consistency across the three cohorts in the magnitude of association of OC insecticides with NHL, but with wide CIs including the null value. Similarly, the magnitude of the mHR of NHL subtypes in association with ever use of OC insecticides was close to the null value and had wide CIs. Exposure to OC insecticides included at least one or a mix of several active ingredients from that group of pesticides, and therefore assessing exposure at the group level may have reflected use or potential use of other active ingredients besides DDT and lindane, which were evaluated individually. However, ever use of DDT and lindane strongly influenced the classification of a cohort member as exposed to the OC insecticides group, particularly in AGRICAN and CNAP. Of farmers and workers classified as ever exposed to OC insecticides, 97% were classified as exposed to lindane and 68% to DDT. These proportions were 89% and 71%, respectively, in CNAP and 36% and 49%, respectively, in AHS.

Ever exposure to DDT and lindane showed associations with NHL of similar magnitude as for the OC group, with no evidence of heterogeneity of effects among the three cohorts, but with CIs including 1. IARC evaluated the carcinogenicity of lindane and DDT in 2015, classifying lindane as Group 1 (carcinogenic to humans) and DDT as Group 2 A (probably carcinogenic to humans).^40^^,^^41^ For lindane, sufficient evidence of carcinogenicity in both humans and experimental animals supported the classification.^40^ For DDT, the IARC evaluation indicated sufficient evidence of carcinogenicity in animals and limited evidence of carcinogenicity in humans. EPA evaluated the carcinogenic potential of lindane in 2001, classifying it as ‘suggestive evidence of carcinogenicity, but not sufficient to assess human carcinogenic potential’ [http://npic.orst.edu/chemicals_evaluated.pdf]. In 2000, EPA classified DDT as Group B2 based on observed occurrence of liver cancer in experimental animals (and ‘inadequate evidence’ or ‘no data’ from epidemiological studies) [http://npic.orst.edu/factsheets/archive/ddttech.pdf].

In our analysis, mHRs for subtypes of NHL with ever use of DDT or lindane showed no association, with magnitudes below 1, except for CLL/SLL, for which the mHR for lindane was 1.06 (95% CI: 0.79–1.41).

Notably, it may be argued that the follow-up period of our study (starting in 2005 in AGRICAN and in 1993 in CNAP and AHS) was after the time period when DDT/lindane-associated risks may have emerged, because DDT was banned in France, Norway and the USA in the early 1970s and lindane was last registered for use in crops in France in 1998, in Norway in 1991 and continued in the USA only with exceptional use until 2006. However, for a lipophilic and persistent pesticide such as DDT, exposure might still be relevant even decades beyond the active period of use, particularly to its metabolite DDE, justifying their inclusion in our analyses. Studies focusing on biomarkers of exposure to OC insecticides in serum, plasma and adipose tissue and risk of NHL have recently been summarized in a meta-analysis^42^ showing no association with DDT (mOR = 1.02, 95% CI: 0.81–1.28; I^2 ^= 0%) while reporting stronger associations with DDE (mOR = 1.38, 95% CI: 1.14–1.66; I^2 ^= 0%) and with other OC pesticides not covered in our analyses. However, capturing exposure to these pesticides may have been more susceptible to misclassification, and therefore application of ever versus never use for risk analyses may have been too crude. The limitation of using this exposure metric is supported by a previous AHS publication with the same follow-up period as ours, which included both private and commercial applicators, reporting no association with ever use of DDT or lindane but finding positive exposure–response trends by total days of exposure both to lindane (*P* = 0.004, 85 cases) and to DDT (*P* = 0.02, 182 exposed cases).^32^ Nevertheless, this does not easily explain why we observed a moderate inverse association for OC insecticides consistently across all three studies, unless it is due to an OC insecticide-associated NHL risk occurring before the start of follow-up, with exposed farmers diagnosed with NHL before the time of enrolment and excluded from entering follow-up. Figure 1 shows the number of farmers/workers with prevalent cancer at enrolment, including cases of NHL, and therefore not included in our analyses. Other explanations may be exposure misclassification and/or chance.

### Phenoxy herbicides

Unlike previously published reports where an increase in NHL risk was suggested, our analysis found inverse associations between exposure to the broader group of phenoxy herbicides and NHL.^16^^,^^39^^,^^43–45^ Active ingredients within this group of herbicides have previously been found to be contaminated with dioxins, including the established human carcinogen 2, 3, 7, 8-tetrachlorodibenzo-*para*-dioxin.^44^^,^^46^ The magnitude of the mHRs of NHL subtypes in association with ever use of phenoxy herbicides was close to the null value and with wide CIs including 1. Exposure to the three active ingredients evaluated within this group of herbicides (2, 4-D, MCPA and MCPP) showed associations with NHL of similar magnitude as that observed for the phenoxy herbicide group, but with CIs including 1. The mHRs for FL in association with 2, 4-D and MCPA were slightly elevated, but with wide CIs including the null value. A recent meta-analysis reported no association of ever exposure to 2, 4-D with NHL (mRR = 0.97, 95% CI: 0.77–1.22; I^2 ^= 29%).^47^

IARC evaluated the carcinogenicity of 2, 4-D in 2015, classifying it as Group 2B (possibly carcinogenic to humans), based on limited evidence in experimental animals for carcinogenicity and inadequate evidence for carcinogenicity in humans.^41^ EPA last evaluated the carcinogenicity of 2, 4-D in 2004, classifying it as Group D (not classifiable as to human carcinogenicity) [http://npic.orst.edu/factsheets/archive/2,4-DTech.html, 4-DTech.html]. The EC does not classify 2, 4-D as carcinogenic [http://ec.europa.eu/food/plant/pesticides/eu-pesticides-database/public/?event=activesubstance.detail&language=EN&selectedID=874].

### Comparison of the three cohorts

Differences among the cohorts may have affected risk estimates. Inclusion criteria for each cohort influenced the prevalence of exposure. AHS enrolled farmers at the time of obtaining or renewing licenses to apply restricted-use pesticides, whereas in AGRICAN and CNAP, identification of cohort members was not tied to potential use of pesticides. Indeed, 67% of AGRICAN and 45% of CNAP cohort members were classified as ever exposed to any of the evaluated pesticides, compared with 99% of AHS cohort members.

In addition, only (potential) use of pesticides on crops was considered in the two cohorts using CEMs to derive pesticide exposure. By design AHS, in addition to pesticides used on crops, also asked farmers about insecticides commonly used on animals. Consequently, the prevalence of a limited number of insecticides (for example, malathion) used in animal husbandry was higher in AHS than in the other two cohorts.

Differences in agricultural practices among the three countries might also have influenced exposure. These differences include agricultural practices in the production of any given crop, crops grown, a crop’s pest pressure and need for pest control, application method, use of protective clothing, worker’s age or a combination of these and other factors.^21–24^ For example in AGRICAN, 45% of farmers reported having cultivated vineyards, a production highly dependent on the use of fungicides, which represent about 80% of all pesticides used in vine production.^48^ Not surprisingly, >60% of farmers/farm workers in AGRICAN were classified as ever users of fungicides, compared with >30% in CNAP and 10% in AHS.

There was evidence of heterogeneity of exposure–outcome effects across the cohorts for some pesticides, as illustrated for esfenvalerate with mHR of 1.65 for MM (I^2 ^= 89%; *P*-value ≤ 0.001). This association was strongest in AHS (HR = 6.98, 95% CI: 2.77–17.59), compared with CNAP (HR = 0.85, 95% CI: 0.60–1.21) and AGRICAN (HR = 0.99, 95% CI: 0.56–1.74). Esfenvalerate was estimated to have been used by 42% of farm owners/farm workers in AGRICAN, 23% in CNAP and 1% in AHS. Among the crops included in our study, esfenvalerate is registered for use in five of seven crops in France and in only two of five crops in Norway.

### Strengths and limitations

The most important strength of our study is the large number of exposed cases. Although the precision of many of the meta-risk estimates was still limited, it was nonetheless much better than for the individual cohorts and therefore provides additional insight into NHL and subtype-specific risks. An additional strength is that data were derived from prospective cohort studies,^22–24^ thus minimizing differential recall bias.

In our analysis, exposure misclassification is probably non-differential, because pesticide exposure was reported or assessed based on information available before the occurrence of the health outcome, introducing bias towards the null and possibly giving rise to false-negative results.^49^ Exposure misclassification may have also reduced the statistical power of our analysis. In addition, given some evidence for an increased NHL risk in farmers^50^^,^^51^ and some evidence that several pesticides may increase risk,^13^^,^^18^ our use of a reference group of never users of a certain pesticide, which included farmers exposed to many other pesticides, is another limitation. The carcinogenicity of several of the pesticides evaluated is unknown; therefore, if they were associated with NHL, we may have underestimated NHL risk in our study by including exposed farmers in the reference group.

Our exposure assessment approach in the two cohorts based on CEMs that relied on crop cultivation and pesticide registration and sales data will have resulted in a lower specificity than when pesticide use is self-reported at the active ingredient level. Registration of an active ingredient and recommendation for use on a given crop, or documentation that the pesticide was sold in the country, do not necessarily mean that it was used by an individual farmer, leading to overestimation of exposure prevalence. Probability of use of pesticides was not available in the CEMs. The magnitude of misclassification may be limited for commonly applied pesticides but could be substantial for rarely used pesticides.^52^ Our analysis demonstrated moderate to high correlations between ever use of active ingredients and between ever use of chemical groups in two cohorts (median correlations 0.63 and 0.64, respectively, in AGRICAN and 0.62 and 0.78, respectively, in CNAP), in contrast to much lower estimates of correlation in AHS (median correlations 0.06 and 0.13, respectively). For instance, between glyphosate and deltamethrin, we observed moderate correlation in AGRICAN (0.71) and CNAP (0.66) and very low correlation in AHS (0.01); between exposure to OC insecticides and phenoxy herbicides, we observed low (0.19), moderate (0.51) and high (0.83) correlations in AHS, AGRICAN and CNAP, respectively (Supplementary Table 4, available as Supplementary data at *IJE* online). The correlations between the active ingredients are a net result of farming practices and the tools we used to assess exposure. High correlation reduces our ability to distinguish independent effects of individual chemical groups or active ingredients.

Further, exposure misclassification may have also arisen from not taking into account re-entry tasks entailing contact with previously applied pesticides (i.e. fruit picking, harvesting), a source of exposure that could be crop- or task-dependent, and as high as or higher than exposure originating from application.^1^^,^^22^ However, re-entry work was not evenly distributed in the cohorts in our study, based on the most frequently reported crops cultivated; for example in the French cohort, 73% of male and 56% of female farm workers performed re-entry work in vineyards, but vineyards were a rarely reported crop in AHS (1%, Table 1).^22^

An additional exposure assessment limitation involves using ever/never use of pesticide active ingredients as a metric of exposure to explore associations with cancer outcomes as a logical first step. This metric alone is not sufficient to characterize cancer risk from pesticide exposure.

Finally, false-positive associations among our findings may have occurred, given the large number of comparisons (14 chemical groups and 33 active ingredients with five cancer outcomes).

## Conclusions

In this combined analysis of >300 000 farmers and agricultural workers from cohorts in France, Norway, and the USA, we found a few moderate associations with ever use of specific chemicals or chemical groups. Among 33 active ingredients evaluated, we observed elevations in risks of NHL overall in association with the organophosphate insecticide terbufos, of CLL/SLL with the pyrethroid insecticide deltamethrin and of DLBCL with the organophosphorus herbicide glyphosate. Among 14 chemical groups, two (OC insecticides and phenoxy herbicides) had inverse associations with NHL overall. Due to low precision for many active ingredient–NHL subtype-specific risk estimates and the expected magnitude of exposure misclassification, associations may also have been missed or attenuated by our approach.

Extensive harmonization of available exposure data from the three independently conducted cohort studies was accomplished successfully. Hence, this analysis represents an important step forward in harmonizing data from large-scale agricultural cohorts from different countries, enabling access to large numbers of cancer cases and in particular exposed cases, which is essential to detect associations between pesticide exposures and cancer. Because NHL subtypes may differ aetiologically with respect to individual pesticide active ingredients and because the number of these ingredients used in agriculture can be sizeable, accrual of larger numbers of cancer cases varying in their exposure to the pesticides under investigation is required. Improvements in the specificity of the exposure assignments, by incorporating probability of use and adding parameters reflecting duration, frequency and intensity of use, are required and planned for future in-depth analysis of the associations reported in our analysis and for the development of the CEMs within the AGRICOH consortium.^53^ Continuation and further endorsement of international collaborations are needed to maintain this important line of research on the carcinogenic effects of pesticide exposure.

## Funding

This work was supported by a grant from the Office National de l’Eau et des Milieux Aquatiques (ONEMA), Plan d’action national ECOPHYTO 2018, Axe 3, Volet 4, France, as part of the 2011 call for research projects of the ANSES Programme on “Environmental and Occupational Health”. In addition, this work was supported in part by the International Agency for Research on Cancer, the Intramural Research Program of the National Cancer Institute, National Institutes of Health (Z01-CP010119), and the Ammodo van Gogh travel grant VGP.14/20.

## Supplementary Material

dyz017_Supplementary_Data
