# Supplementary material for: Pesticide use and risk of non-Hodgkin lymphoid malignancies in agricultural cohorts from France, Norway and the USA: a pooled analysis from the AGRICOH consortium
Source: Int J Epidemiol. 2019 Mar 18;48(5):1519–35. doi: 10.1093/ije/dyz017 (PMC6857760; doi:10.1093/ije/dyz017)
Supplement: dyz017_Supplementary_Data [file dyz017_supplementary_data.zip › dyz017-Suppl_data/Supplementary_Table_3.pdf]

Supplementary Table 3. Ever use of 14 pesticide chemical groups and 33 active ingredients and meta-risk estimates, minimally-adjusted, of Non-Hodgkin lymphoid malignancies diagnosed during follow-up in farmers and farm workers in three cohort studies from France, Norway and the USA

	Non-Hodgkin lymphoid malignancies (NHL)				Chronic lymphocytic leukaemia/small lymphocytic lymphoma (CLL)				Diffused large B-cell lymphoma (DLBCL)			
	N (a)	HR(b)	95% CI	I^2	N (a)	HR(b)	95% CI	I^2	N (a)	HR(b)	95% CI	I^2
OP INSECTICIDES	1417	1.02	(0.83- 1.26)	65%	318	1.31	(1.01- 1.69)	10%	256	0.96	(0.77- 1.19)	0%
. Chlorpyrifos	453	0.97(d)	(0.83- 1.13)	16%	106	1.02(d)	(0.76- 1.36)	0%	86	0.88(d)	(0.65- 1.20)	0%
. Dichlorvos	523	1.07	(0.90- 1.26)	45%	116	1.13	(0.76- 1.68)	50%	81	0.89	(0.69- 1.16)	0%
. Malathion	1208	1.01	(0.84- 1.22)	68%	274	1.32	(1.08- 1.61)	0%	209	0.84	(0.60- 1.17)	54%
. Parathion	995	0.99	(0.81- 1.21)	72%	210	1.18	(0.90- 1.55)	29%	162	0.93	(0.75- 1.15)	0%
. Terbufos	300	1.10(d)	(0.87- 1.40)	57%	76	1.19(d)	(0.87- 1.62)	0%	56	0.90(d)	(0.55- 1.47)	44%
CARBAMATE INSECTICIDES	1254	1.02	(0.85- 1.22)	69%	283	1.27	(0.95- 1.70)	40%	219	0.90	(0.71- 1.15)	16%
. Aldicarb	526	1.06	(0.90- 1.26)	46%	116	1.33	(1.05- 1.69)	0%	82	0.86	(0.66- 1.13)	4%
. Carbaryl	651	0.95	(0.83- 1.08)	0%	148	1.02	(0.76- 1.37)	0%	125	0.81	(0.61- 1.09)	0%
. Carbofuran	254	0.99(d)	(0.85- 1.16)	0%	63	1.06(d)	(0.77- 1.46)	0%	44	0.74(d)	(0.51- 1.07)	0%
. Pirimicarb	796	1.01(c)	(0.75- 1.37)	83%	176	1.26(c)	(0.74- 2.14)	75%	123	0.89(c)	(0.58- 1.38)	52%
OC INSECTICIDES	1258	0.98	(0.81- 1.19)	69%	288	1.22	(0.91- 1.65)	44%	215	0.80	(0.53- 1.22)	68%
. DDT	976	1.03	(0.92- 1.14)	20%	220	1.21	(0.99- 1.48)	2%	165	0.90	(0.72- 1.12)	0%
. Lindane	953	0.99	(0.83- 1.18)	65%	217	1.17	(0.79- 1.73)	68%	160	0.90	(0.65- 1.26)	46%
PYRETHROID INSECTICIDES	934	1.08	(0.90- 1.29)	68%	211	1.30	(0.93- 1.81)	52%	153	0.96	(0.74- 1.25)	23%
. Deltamethrin	627	1.04(c)	(0.74- 1.47)	86%	148	1.41(c)	(0.82- 2.44)	73%	99	0.94(c)	(0.59- 1.51)	60%
. Esfenvalerate	549	1.18	(0.82- 1.70)	80%	124	1.24(c)	(0.62- 2.50)	81%	88	1.05(c)	(0.76- 1.46)	19%
. Permethrin	812	1.07	(0.91- 1.26)	47%	182	1.27	(0.87- 1.85)	54%	131	0.98	(0.78- 1.23)	0%
(PHENYL) UREA HERBICIDES	979	1.04	(0.88- 1.25)	61%	209	1.32	(1.08- 1.63)	0%	156	0.93	(0.74- 1.17)	6%
. Isoproturon	453	1.10(c)	(0.99- 1.23)	0%	109	1.44(c)	(0.94- 2.21)	51%	73	1.03(c)	(0.78- 1.36)	0%
. Linuron	961	1.07	(0.89- 1.27)	55%	204	1.28	(0.99- 1.65)	15%	155	0.98	(0.78- 1.21)	0%
DICAMBA	815	1.07	(0.95- 1.21)	25%	180	1.11	(0.89- 1.38)	0%	142	0.95	(0.72- 1.26)	19%
CHLOROACETANILIDES	735	1.05	(0.94- 1.17)	0%	161	1.00	(0.79- 1.28)	0%	145	0.98	(0.66- 1.45)	52%
. Alachlor	380	0.96(d)	(0.82- 1.12)	0%	85	0.82(d)	(0.56- 1.20)	20%	74	0.79(d)	(0.57- 1.10)	0%
. Metolachlor	358	0.98(d)	(0.84- 1.14)	0%	88	0.98(d)	(0.72- 1.34)	0%	71	0.84(d)	(0.58- 1.21)	0%
DINITROANILINE HERBICIDES	506	0.93(d)	(0.80- 1.09)	0%	126	1.01(d)	(0.73- 1.39)	0%	101	0.78(d)	(0.56- 1.09)	0%
. Trifluralin	368	0.92(d)	(0.79- 1.08)	0%	90	0.89(d)	(0.64- 1.24)	0%	72	0.80(d)	(0.57- 1.14)	0%
GLYPHOSATE	1131	0.98	(0.76- 1.25)	80%	252	1.09	(0.70- 1.70)	71%	221	1.12	(0.86- 1.45)	21%

PHENOXY HERBICIDES	1204	0.97	(0.80- 1.17)	67%	272	1.07	(0.70- 1.64)	65%	218	0.92	(0.70- 1.21)	27%
. 2,4-D	1167	0.99	(0.82- 1.19)	67%	262	1.03	(0.64- 1.68)	76%	212	0.95	(0.71- 1.26)	34%
. MCPA	774	1.02(c)	(0.83- 1.26)	67%	162	1.18(c)	(0.72- 1.93)	63%	125	0.92(c)	(0.60- 1.42)	50%
. MCPP	776	1.09	(0.99- 1.20)	0%	161	1.28(c)	(0.89- 1.84)	37%	123	0.92(c)	(0.60- 1.42)	51%
THIOCARBAMATE HERBICIDES	1015	1.00	(0.80- 1.24)	78%	225	1.07	(0.67- 1.72)	81%	171	0.91	(0.67- 1.23)	40%
. Butylate	265	0.98(d)	(0.84- 1.14)	0%	60	0.87(d)	(0.62- 1.23)	0%	48	0.78(d)	(0.55- 1.11)	0%
. EPTC	606	1.07	(0.91- 1.26)	51%	133	1.12	(0.77- 1.65)	59%	101	0.98	(0.74- 1.31)	21%
TRIAZINE HERBICIDES	1165	1.02	(0.89- 1.17)	39%	269	1.11	(0.72- 1.71)	66%	212	0.96	(0.78- 1.19)	0%
. Atrazine	546	0.94(d)	(0.79- 1.12)	0%	131	0.91(d)	(0.62- 1.33)	0%	109	0.76(d)	(0.48- 1.20)	37%
. Simazine	303	0.92	(0.79- 1.07)	0%	63	0.91	(0.62- 1.33)	0%	54	0.83	(0.55- 1.25)	24%
TRIAZINONE HERBICIDES	971	1.07	(0.90- 1.27)	62%	215	1.26	(1.03- 1.53)	0%	157	0.92	(0.74- 1.13)	0%
. Metribuzin	971	1.07	(0.90- 1.27)	61%	215	1.26	(1.03- 1.53)	0%	157	0.92	(0.74- 1.13)	0%
DITHIOCARBAMATE FUNGICIDES	961	1.02	(0.84- 1.22)	59%	196	1.17	(0.92- 1.49)	9%	157	0.94	(0.75- 1.17)	0%
. Mancozeb	932	0.99	(0.80- 1.22)	67%	193	1.23	(0.99- 1.52)	0%	149	0.90	(0.72- 1.13)	0%
. Thiram	263	0.90(c)	(0.76- 1.06)	0%	54	0.88(c)	(0.59- 1.33)	0%	42	0.75(c)	(0.42- 1.35)	57%
PHTHALIMIDE FUNGICIDES	921	1.04	(0.87- 1.24)	56%	193	1.25	(1.01- 1.54)	0%	144	0.91	(0.73- 1.14)	0%
. Captafol	800	1.05(c)	(0.80- 1.38)	76%	162	1.15(c)	(0.74- 1.78)	52%	121	0.90(c)	(0.70- 1.14)	0%
. Captan	481	1.03	(0.89- 1.18)	27%	107	1.21	(0.96- 1.52)	0%	74	0.89	(0.68- 1.16)	0%
ARSENICALS	272	0.93(d)	(0.77- 1.13)	0%	60	0.99(d)	(0.64- 1.53)	0%	52	1.02(d)	(0.63- 1.66)	0%

Supplementary Table 3. Ever use of 14 pesticide chemical groups and 33 active ingredients and meta-risk estimates, minimally-adjusted, of Non-Hodgkin lymphoid malignancies diagnosed during follow-up in farmers and farm workers in three cohort studies from France, Norway and the USA (cont.)

	Follicular Lymphoma (FL)				Multiple myeloma/plasma-cell leukaemia (MM)			
	N (a)	HR(b)	95% CI	I^2	N (a)	HR(b)	95% CI	I^2
OP INSECTICIDES	131	1.01	(0.72- 1.41)	0%	313	1.06	(0.88- 1.28)	0%
. Chlorpyrifos	46	1.07(d)	(0.69- 1.67)	0%	97	0.99(d)	(0.71- 1.38)	0%
. Dichlorvos	40	1.05	(0.73- 1.53)	0%	111	1.02	(0.79- 1.32)	14%
. Malathion	114	1.12	(0.83- 1.53)	0%	269	1.08	(0.90- 1.29)	0%
. Parathion	83	1.12	(0.82- 1.52)	0%	230	1.00	(0.77- 1.30)	29%
. Terbufos	35	1.30(d)	(0.80- 2.09)	0%	55	1.06(d)	(0.75- 1.51)	0%
CARBAMATE INSECTICIDES	115	1.04	(0.77- 1.42)	0%	290	1.13	(0.94- 1.36)	0%
. Aldicarb	38	0.95	(0.64- 1.41)	0%	113	0.97	(0.77- 1.21)	0%
. Carbaryl	65	1.28(d)	(0.76- 2.18)	10%	152	1.09	(0.73- 1.62)	42%
. Carbofuran	26	0.98(d)	(0.59- 1.63)	0%	57	1.18(d)	(0.84- 1.65)	0%
. Pirimicarb	58	0.99(c)	(0.70- 1.41)	0%	185	1.06(c)	(0.87- 1.29)	0%
OC INSECTICIDES	114	1.04	(0.77- 1.41)	0%	290	1.08	(0.90- 1.30)	0%
. DDT	79	0.95	(0.67- 1.34)	13%	230	1.05	(0.87- 1.27)	0%
. Lindane	78	1.03	(0.76- 1.39)	0%	216	1.02	(0.84- 1.23)	0%
PYRETHROID INSECTICIDES	81	1.15	(0.85- 1.54)	0%	210	1.09	(0.90- 1.31)	0%
. Deltamethrin	46	1.06(c)	(0.72- 1.56)	0%	135	0.99(c)	(0.80- 1.23)	0%
. Esfenvalerate	40	1.05(c)	(0.72- 1.53)	0%	120	1.63	(0.72- 3.68)	87%
. Permethrin	60	0.96	(0.66- 1.40)	10%	187	1.12	(0.93- 1.36)	0%
(PHENYL) UREA HERBICIDES	81	1.10	(0.81- 1.51)	0%	227	1.09	(0.91- 1.31)	0%
. Isoproturon	28	0.87(c)	(0.57- 1.33)	0%	100	1.01(c)	(0.80- 1.28)	0%
. Linuron	79	1.11	(0.81- 1.52)	0%	224	1.11	(0.92- 1.34)	0%
DICAMBA	73	0.93	(0.67- 1.30)	0%	179	1.16	(0.94- 1.42)	0%
CHLOROACETANILIDES	78	1.07	(0.74- 1.54)	0%	147	0.98	(0.78- 1.24)	0%
. Alachlor	43	0.97(d)	(0.58- 1.62)	0%	78	1.07(d)	(0.77- 1.49)	0%
. Metolachlor	43	1.11(d)	(0.66- 1.88)	0%	71	1.07(d)	(0.76- 1.51)	0%
DINITROANILINE HERBICIDES	57	1.07(d)	(0.64- 1.79)	0%	95	0.85(d)	(0.50- 1.45)	64%
. Trifluralin	39	0.89(d)	(0.54- 1.46)	0%	70	0.94(d)	(0.67- 1.34)	0%
GLYPHOSATE	105	0.95	(0.70- 1.29)	0%	240	1.00	(0.83- 1.21)	0%
PHENOXY HERBICIDES	115	1.06	(0.78- 1.45)	0%	257	0.97	(0.81- 1.17)	0%

. 2,4-D	113	1.10	(0.81- 1.51)	0%	250	1.01	(0.84- 1.22)	0%
. MCPA	60	1.08(c)	(0.76- 1.53)	0%	174	0.98(c)	(0.80- 1.20)	0%
. MCPP	58	1.04(c)	(0.73- 1.49)	0%	174	1.01(c)	(0.83- 1.24)	0%
THIOCARBAMATE HERBICIDES	88	0.98	(0.73- 1.31)	0%	225	1.07	(0.90- 1.29)	0%
. Butylate	31	1.13(d)	(0.70- 1.83)	0%	54	1.05(d)	(0.74- 1.48)	0%
. EPTC	49	0.92	(0.51- 1.65)	53%	138	1.20	(0.98- 1.47)	0%
TRIAZINE HERBICIDES	115	1.17	(0.85- 1.62)	0%	246	0.97	(0.80- 1.18)	0%
. Atrazine	65	1.33(d)	(0.76- 2.31)	0%	108	1.00(d)	(0.68- 1.46)	0%
. Simazine	23	0.94	(0.53- 1.64)	0%	66	0.86	(0.57- 1.30)	30%
TRIAZINONE HERBICIDES	86	1.05	(0.78- 1.42)	0%	221	1.18	(0.98- 1.42)	0%
. Metribuzin	86	1.05	(0.78- 1.42)	0%	221	1.18	(0.98- 1.42)	0%
DITHIOCARBAMATE FUNGICIDES	75	1.03	(0.74- 1.42)	0%	229	1.10	(0.90- 1.33)	0%
. Mancozeb	71	0.99	(0.72- 1.38)	0%	222	1.08	(0.89- 1.31)	0%
. Thiram	17	0.89(c)	(0.47- 1.70)	0%	58	0.81(c)	(0.44- 1.51)	63%
PHTHALIMIDE FUNGICIDES	71	1.03	(0.75- 1.43)	0%	216	1.10	(0.91- 1.33)	0%
. Captafol	62	1.14(c)	(0.80- 1.61)	0%	192	1.15(c)	(0.94- 1.41)	0%
. Captan	36	0.96	(0.65- 1.40)	0%	97	0.88	(0.70- 1.11)	0%
ARSENICALS	17				65	0.91(d)	(0.60- 1.37)	0%

(a) Number of cancer cases
(b) Hazard Ratio: Random effects meta-analysis (minimally adjusted models)
(c) Meta-analysis based on AGRICAN and CNAP only
(d) Meta-analysis based on AGRICAN and AHS only
I^2 I-squared (variation in meta estimate attributable to heterogeneity)
AGRICAN: Cox Regression adjusted for: sex, livestock, retirement status
CNAP: Cox Regression adjusted for: sex, livestock
AHS: Cox Regression adjusted for: sex, state, livestock
mHR estimate for FL in association with arsenicals not available because an estimate could be computed in only one (AGRICAN) of three cohorts.
