# Supplementary material for: Pesticide use and risk of non-Hodgkin lymphoid malignancies in agricultural cohorts from France, Norway and the USA: a pooled analysis from the AGRICOH consortium
Source: Int J Epidemiol. 2019 Mar 18;48(5):1519–35. doi: 10.1093/ije/dyz017 (PMC6857760; doi:10.1093/ije/dyz017)
Supplement: dyz017_Supplementary_Data [file dyz017_supplementary_data.zip › dyz017-Suppl_data/Supplementary_Data2.doc]

**Supplementary Figure 1.** Prevalence of reported/estimated ever use of pesticide chemical groups and active chemical ingredients by cohort for (a) insecticides, (b) herbicides, (c) fungicides, arsenicals, and for any pesticide.

(a)

(b)

(c)
