# Supplementary material for: Pesticide use and risk of non-Hodgkin lymphoid malignancies in agricultural cohorts from France, Norway and the USA: a pooled analysis from the AGRICOH consortium
Source: Int J Epidemiol. 2019 Mar 18;48(5):1519–35. doi: 10.1093/ije/dyz017 (PMC6857760; doi:10.1093/ije/dyz017)
Supplement: dyz017_Supplementary_Data [file dyz017_supplementary_data.zip › dyz017-Suppl_data/Supplementary_Data1.doc]

**Supplementary Table 1.** Pesticide active ingredients considered within the selected chemical groups to assign ever exposure to corresponding chemical group by cohort (adapted from Brouwer et al., 2016^21^)

**Supplementary Table 2.** Distribution of lymphoid malignancies diagnosed during follow-up in the combined study population and by cohort

|  | Combined population | | AGRICAN | | CNAP | | AHS | |
| --- | --- | --- | --- | --- | --- | --- | --- | --- |
|  | N | % | N | % | N | % | N | % |
| LYMPHOID MALIGNANCIES | 2545 | 100.0% | 454 | 100.0% | 1567 | 100.0% | 524 | 100.0% |
| __ Hodgkin lymphoma | 91 | 3.5% | 15 | 3.3% | 57 | 3.6% | 19 | 3.6% |
| ____ Classical Hodgkin lymphoma | 80 | 3.1% | 15 | 3.3% | 47 | 2.9% | 18 | 3.4% |
| ____ Nodular lymphocyte predominant Hodgkin lymphoma | 11 | 0.4% | . | . | 10 | 0.6% | 1 | 0.1% |
| __ Non-Hodgkin lymphoma (NHL)* | 2430 | 95.4% | 439 | 96.6% | 1498 | 95.5% | 493 | 94.0% |
| ____ Non-Hodgkin lymphoma, B-cell | 2258 | 88.7% | 396 | 87.2% | 1396 | 89.0% | 466 | 88.9% |
| ______ Precursor non-Hodgkin lymphoma, B-cell | 18 | 0.7% | 4 | 0.8% | 12 | 0.7% | 2 | 0.3% |
| ______ Mature non-Hodgkin lymphoma, B-cell | 2106 | 82.7% | 392 | 86.3% | 1262 | 80.5% | 452 | 86.2% |
| ________ Chronic/small/prolymphocytic/mantle B-cell NHL | 596 | 23.4% | 104 | 22.9% | 348 | 22.2% | 144 | 27.4% |
| _________ Chronic/small lymphocytic leukemia/lymphoma (CLL/SLL)* | 497 | 19.5% | 94 | 20.7% | 280 | 17.8% | 123 | 23.4% |
| _________ Prolymphocytic leukemia, B-cell | 2 | 0.0% | 1 | 0.2% | 1 | 0.0% | . | . |
| _________ Mantle-cell lymphoma | 97 | 3.8% | 9 | 1.9% | 67 | 4.2% | 21 | 4.0% |
| ________ Lymphoplasmacytic lymphoma/Waldenström | 136 | 5.3% | 38 | 8.3% | 90 | 5.7% | 8 | 1.5% |
| ________ Diffuse large B-cell lymphoma (DLBCL)* | 434 | 17.0% | 75 | 16.5% | 246 | 15.6% | 113 | 21.5% |
| ________ Burkitt lymphoma/leukemia | 16 | 0.6% | 3 | 0.6% | 12 | 0.7% | 1 | 0.1% |
| ________ Marginal-zone lymphoma (MZL) | 83 | 3.2% | 27 | 5.9% | 43 | 2.7% | 13 | 2.4% |
| ________ Follicular lymphoma (FL) | 214 | 8.4% | 34 | 7.4% | 116 | 7.4% | 64 | 12.2% |
| ________ Hairy-cell leukemia | 30 | 1.1% | 3 | 0.6% | 21 | 1.3% | 6 | 1.1% |
| ________ Plasma cell neoplasms | 597 | 23.4% | 108 | 23.7% | 386 | 24.6% | 103 | 19.6% |
| __________ Plasmacytoma | 36 | 1.4% | 5 | 1.1% | 24 | 1.5% | 7 | 1.3% |
| __________ Multiple myeloma/plasma-cell leukemia (MM)* | 561 | 22.0% | 103 | 22.6% | 362 | 23.1% | 96 | 18.3% |
| ______ Non-Hodgkin lymphoma, B-cell, NOS | 134 | 5.2% | . | . | 122 | 7.7% | 12 | 2.2% |
| ____ Non-Hodgkin lymphoma, T-cell | 120 | 4.7% | 25 | 5.5% | 77 | 4.9% | 18 | 3.4% |
| ______ Precursor non-Hodgkin lymphoma, T-cell | 5 | 0.1% | 2 | 0.4% | 2 | 0.1% | 1 | 0.1% |
| ______ Mature non-Hodgkin lymphoma, T-cell | 115 | 4.6% | 23 | 5.1% | 75 | 4.8% | 17 | 3.3% |
| ____ Non-Hodgkin lymphoma, unknown lineage | 52 | 2.0% | 18 | 3.9% | 25 | 1.5% | 9 | 1.7% |
| __ Composite Hodgkin lymphoma and NHL | 1 | 0.0% | . | . | 1 | 0.0% | . | . |
| __ Lymphoid neoplasm, NOS | 23 | 0.9% | . | . | 11 | 0.7% | 12 | 2.2% |

* Lymphoid entities for which results are provided in this analysis.

**Supplementary Table 4.** Pearson correlation coefficients between exposure to a selection of pesticide chemical groups and active ingredients (ever use) by cohort study.

|  | **AGRICAN** | | | | |
| --- | --- | --- | --- | --- | --- |
|  | Terbufos | Deltamethrin | Glyphosate | Lindane | DDT |
| Terbufos | 1.00 | 0.50 | 0.58 | 0.39 | 0.17 |
| Deltamethrin | | 1.00 | 0.71 | 0.70 | 0.33 |
| Glyphosate | |  | 1.00 | 0.51 | 0.19 |
| Lindane |  |  |  | 1.00 | 0.67 |
| DDT |  |  |  |  | 1.00 |
|  | **CNAP** | | | | |
| Terbufos | 1.00 | Na | n.a. | n.a. | Na |
| Deltamethrin | | 1.00 | 0.66 | 0.68 | 0.49 |
| Glyphosate | |  | 1.00 | 0.72 | 0.62 |
| Lindane |  |  |  | 1.00 | 0.65 |
| DDT |  |  |  |  | 1.00 |
|  | **AHS** | | | | |
| Terbufos | 1.00 | 0.01 | 0.09 | 0.10 | 0.01 |
| Deltamethrin | | 1.00 | 0.01 | 0.00 | -0.01 |
| Glyphosate | |  | 1.00 | 0.10 | 0.06 |
| Lindane |  |  |  | 1.00 | 0.16 |
| DDT |  |  |  |  | 1.00 |

n.a. Not applicable

|  | OC Insecticides | | |
| --- | --- | --- | --- |
|  | AGRICAN | CNAP | AHS |
| Phenoxy Herbicides | 0.51 | 0.83 | 0.19 |
